# Investigating the impact of oral health status on the prevalence of falls among caregivers and non‐caregivers in the United States: A retrospective study

**DOI:** 10.1111/scd.13097

**Published:** 2024-12-21

**Authors:** Mohammed Alshanbari, Erin Bouldin, Hesham Alhazmi, Kenneth Knapp

**Affiliations:** ^1^ Department of Health Management and Medical Informatics Health Sciences College at Al‐Leith Umm Al‐Qura University Makkah Saudi Arabia; ^2^ Department of Internal Medicine, Division of Epidemiology Utah University, Salt Lake City Utah USA; ^3^ Department of Veterans Affairs Medical Center Health Systems Research Salt Lake City Utah USA; ^4^ Department of Preventive Dentistry Faculty of Dentistry Division of Pediatric Dentistry Umm Al‐Qura University Makkah Saudi Arabia; ^5^ Department of Public Health School of Health Sciences and Practice New York Medical College Valhalla New York USA

**Keywords:** caregivers, dental utilization, oral health, prevalence of falls, teeth lost

## Abstract

**Objective:**

Examining the association between oral health and the prevalence of falls among US caregivers.

**Background:**

There is a scarcity of research on the association between oral health status and the prevalence of falls among older adults and caregivers.

**Materials and methods:**

Data from the Behavioral Risk Factor Surveillance System (BRFSS) surveys from 2016 to 2020 were analyzed. The analysis was limited to individuals aged 45 years and older (*n* = 97 550). The outcome variable was if a person had fallen at least once during the past 12 months. The main predictor variables were the number of teeth removed, the last dentist visit, and caregiver status. Data were analyzed using bivariate descriptive statistics, chi‐square tests, and logistic regression to estimate adjusted odds ratios (AORs).

**Results:**

Having one to five teeth removed was linked to an increased prevalence of falls [AOR: 1.13, 95% confidence interval (95% CI): 1.02–1.25, *p* = .017] and was highest for complete tooth loss (AOR: 1.35, 95% CI: 1.15–1.58, *p* < .001). Caregivers had increased fall odds (AOR: 1.23, 95% CI: 1.10–1.38, *p* < .001). Caregivers’ prevalence of fall increases with six or more teeth removed (AOR: 1.41, 95% CI: 1.02–1.94, *p* = .038) and all teeth removed (AOR: 1.47, 95% CI: 1.03–2.09, *p* = .030). Dental visits in the past year were associated with a 21% reduced fall risk for caregivers (AOR = 0.79, 95% CI: 0.64–0.98, *p* = .035).

**Conclusion:**

The study showed that maintaining good oral health and utilizing dental services may be associated with lower fall risks, particularly among caregivers, suggesting the need for integrating oral health into fall prevention strategies.

## 1 INTRODUCTION

Falls are regarded as the most common cause of non‐fatal injuries that are treated in hospital emergency rooms.^1^ Each year, approximately 28% of adults aged 65 years and older report falling. The definition of a fall is “an event which results in a person coming to rest inadvertently on the ground, floor, or other lower level.”^2^ In the United States, older adults risk serious injuries due to impairments in balance, vision, and cognition; comorbidities; polypharmacy; physical inactivity; and environmental hazards.^3^ Additionally, oral health problems are more prevalent among the older population.^4^, ^5^ A study in 2012 reported an association between a low number of teeth and a higher frequency of falls: those with fewer than 19 teeth had a significantly higher risk of falling than those with 20 or more teeth.^6^ Another study revealed a statistically significant association between the number of teeth present and the risk of falling.^7^ Furthermore, it was found that people with natural teeth had better balance than those without teeth. This research suggests that the periodontal ligament's proprioceptive input is integral to postural equilibrium.^8^ However, there is no definitive evidence directly correlating diminished oral proprioception due to periodontal ligament loss with gait instability in the elderly. Nevertheless, studies by Kohli et al.^7^ and Gangloff et al.^9^ implicate the trigeminal nerve's sensory innervation and dental occlusion as contributory factors to balance modulation, indicating a complex interplay between oral sensory mechanisms and postural control. Further, a study by Brand et al. reported that teeth lost among healthy older individuals can decrease their walking speed, which may negatively impact their stability while walking.^10^

Due to the growing proportion of adults aged 65 years and older, the demand for caregiving is increasing.^11^ Around 34.2 million people have provided care to adults aged 50 years or older.^12^ Caregiving is considered a major public health issue because caregivers play an important role in enabling older adults and people living with disabilities to remain in the community and because providing care can have negative physical and mental health implications on caregivers.^13^, ^14^, ^15^ A study of caregivers conducted by Wu et al. found that low‐income caregivers were less likely to seek preventive dental care. Also, caregivers who had lost six or more teeth or had completely lost all their permanent teeth were less likely to have had a dentist visit in the past year and bad oral health.^16^ Another study that used data from the 2016 Behavioral Risk Factor Surveillance System (BRFSS) reported that caregivers had worse oral health status, defined as one or more teeth were removed due to decay, gum disease, or infection, than non‐caregivers.^17^

There is a scarcity of research on the association between oral health status and the prevalence of falls among older adults and caregivers in the United States. This study investigated the potential association between oral health status, dental service utilization, and the likelihood of falls among caregivers and non‐caregivers, and it evaluated the relationship between oral health and falls among caregivers. We hypothesized that individuals and caregivers aged 45 years and older with poor oral health status and those who did not use oral health services regularly would experience more falls than individuals and caregivers with better oral health status and service use. We also hypothesized that caregivers would experience more falls than non‐caregivers.

## 2 METHODS

This cross‐sectional study used de‐identified secondary data from the 2016, 2018, and 2020 BRFSS surveys, available as public use files on the Centers for Disease Control and Prevention (CDC) website (https://www.cdc.gov/brfss). Participants are randomly selected community‐dwelling U.S. residents aged 18 years and older. All BRFSS respondents aged 45 years and older are asked about their experience of falls. The Caregiver module is an optional module that states can choose to use. This optional module provides specific insights into caregivers' demographics and health‐related behaviors, offering valuable data for analyzing their risk factors, including falls. Therefore, our study included individuals aged 45 years and older from the 24 U.S. states and territories that included the Caregiver module in one of the study years. The analysis included 97 550 respondents.

### 2.1 Falls

The primary outcome variable is whether or not a person had a fall at least once in the past 12 months. Information on falls was acquired through the following question from BRFSS questionnaire: “In the past 12months, how many times have you fallen?”.

To construct the outcome variable indicating whether a person had fallen ever versus never, we categorized respondents as having fallen at least one time in the past year (response: one or more falls) versus never (response: zero falls), following Ylitalo and Karvonen‐Gutierrez.^18^

### 2.2 Caregiving

The BRFSS asked the following question to ascertain the respondent's caregiving status: “During the past 30 days, did you provide regular care or assistance to a friend or family member who has a health problem or disability?” The responses were (1) yes; (2) no; (7) don't know/not sure; (8) caregiving recipient died in the past 30 days; (9) refused. This variable was recoded into two categories: (1) caregivers, and (0) non‐caregivers, which included people who answered “no” and “caregiving recipient died in the past 30 days.” BRFSS respondents who answered “don't know/not sure” or “refused” or had missing responses at random were excluded from the data analyses.

### 2.3 Oral health variables

#### 2.3.1 Oral health status (number of permanent teeth removed)

The BRFSS survey asked the following question to ascertain the respondent's loss of teeth due to dental decay or periodontal diseases: “How many of your permanent teeth have been removed because of tooth decay or gum disease? Include teeth lost to infection, but do not include teeth lost for other reasons, such as injury or orthodontics.” The question was categorized according to Wiener and Sambamoorthi as 0 = no missing teeth, 1 = 1–5 teeth removed, 2 = 6 or more, but not all teeth removed, 3 = no teeth.^19^

#### 2.3.2 Dental services utilization

The BRFSS questionnaire asked the following questions to assess use of dental services: “Including all types of dentists, such as orthodontists, oral surgeons, and all other dental specialists, as well as dental hygienists, how long has it been since you last visited a dentist or a dental clinic for any reason?” We classified respondents into two categories based on the American Dental Association's guidelines: “Yes” for those who visited a dentist within the past 12 months, and “No” for those who visited a dentist more than 12 months ago. The “No” category includes various subgroups indicating the length of time since their last visit: within the past 2 years, between 1 and 2 years ago, within the past 5 years, between 2 and 5 years ago, over 5 years ago, or never. We classified responses based on participants’ self‐reports of visiting a dentist at least once in the past 12 months.^20^

### 2.4 Covariates

Age, sex, marital status, race/ethnicity, education, employment status, physical activity, smoking, alcohol drinking history, body mass index (BMI), number of chronic diseases, and depression were used as covariates in our study. Race/ethnicity was grouped into non‐Hispanic Black, non‐Hispanic White, Hispanic, and non‐Hispanic other race (including multiracial, American Indian or Alaskan Native only, and Other race only). Education level had four categories: no high school, high school education, attended college or some college or technical school, and college graduate.^21^ Employment status had two categories: employed and not employed.^22^ Physical activity was based on if participants performed any physical activities or exercises during the past month or not.^23^ Smoking groups were never smoked, current smoker, and former smoker. Alcohol drinking history included those who were heavy drinkers (adult men having more than 14 drinks per week and adult women having more than 7 drinks per week) or not. BMI was categorized into underweight (below 18.5), normal weight (18.5–24.), overweight (25.0–29.9), and obese (30.0 and above).^18^ Number of chronic diseases was based on whether a healthcare professional ever told the participants they had diabetes mellitus, cardiovascular disease, arthritis, cancer, asthma, or chronic obstructive pulmonary disease (COPD). We created a variable to classify respondents as having no chronic condition, one chronic condition, or two or more chronic conditions. Depression was categorized as if having ever been diagnosed with a depressive disorder (including depression, major depression, dysthymia, or minor depression) or not.^18^

### 2.5 Statistical analysis

Descriptive analyses included calculating percentages for all variables. Bivariate analyses involved examining relationships between fall prevalence, oral health status, last dental visit, caregiving status, and sociodemographic factors using chi‐square tests. Binary logistic regression models were then applied to assess associations between oral health status, dental services utilization, caregiving status, and falls among all respondents and then among caregivers aged 45 years and older, while adjusting for socio‐demographic factors, physical activity, smoking, alcohol drinking history, BMI, chronic diseases, and depression. The study used two separate logistic regression models to investigate these associations, with outcomes categorized as either experiencing a fall at least once in the last 12 months (yes) or not (no). The first model focused on these aspects adjusted for caregiving status and using sociodemographic, health, and lifestyle variables among 45 years and older. The second model investigated the impact of oral health and fall adjusted for the same covariates among caregivers 45 years and older to determine the impact of oral health status on fall risk, especially among caregivers. The analysis was conducted using Stata version 16.1 software (StataCorp, CollegeStation, Texas, USA), and results included adjusted odds ratios (AORs), 95% confidence intervals (95% CIs), and *p*‐values for each variable included in the models. All analyses accounted for the complex sampling design of the BRFSS by using svy statements and incorporating the primary sampling unit, stratum, and weight information.^24^ We considered differences statistically significant if *p* < .05.

## 3 RESULTS

### 3.1

#### 3.1.1 Overall sociodemographic characteristics, health behaviors, and oral health status

There were 97 550 respondents in total. The majority were between the ages of 45–64 years (58.4%), non‐Hispanic White (72%), married (61%), and had completed high school (31%) (Table 1). Approximately 57% of them were unemployed, roughly 68% had exercised in the last month, and 53% of them had never smoked. Moreover, about 83% of respondents had encountered depressive disorders, while almost 37% were overweight and 34% had two or more chronic conditions. With regard to reporting falls, approximately 26% of individuals aged 45 years and older had experienced at least one fall in the past year. Regarding dental services utilization and oral health, 65% of respondents had used dental services over the last year. Additionally, the majority of participants, 38.5%, had all their teeth.

**TABLE 1:** Demographic characteristics, behaviors, chronic conditions, falls, number of permanent teeth removed, and utilization of dental services of 45+ and caregiver status who participated in BRFSS surveys, United States, 2016, 2018, and 2020.

		Caregiving status		
Variables	Total number of respondents (*N* = 97 550)^a^ *N* (Weighted %)^b^	Caregiver (*N* = 20 089)^a^ *N* (Weighted %)^b^	Non‐caregivers (*N* = 77 461)^a^ *N* (Weighted %)^b^	Chi‐square *p*
Age (years)				
45–64	48 696 (58.42)	(65.40)	(56.53)	<.001
65 and older	48 854 (41.57)	(34.59)	(43.46)	
Sex				
Male	45 437 (50.84)	(49.95)	(51.08)	.3267
Female	52 106 (49.15)	(50.04)	(48.91)	
Ethnicity				
Non‐Hispanic White	76 757 (72.30)	(75.22)	(71.50)	.0040
Non‐Hispanic Black	6844 (11.07)	(11.36)	(10.99)	
Hispanic	8049 (12.41)	(9.53)	(13.20)	
Other race Non‐Hispanic	4218 (4.20)	(3.86)	(4.29)	
Marital status				
Not married	42 063 (38.97)	(34.65)	(40.15)	<.001
Married	54 860 (61.02)	(65.34)	(59.84)	
Education				
No high school	7764 (13.64)	(10.01)	(14.63)	<.001
High school education	27 074 (30.69)	(30.08)	(30.86)	
Attended college or some college or technical school	26 782 (28.86)	(33.46)	(27.60)	
College graduate	26 782 (27.79)	(26.42)	(26.89)	
Employment status				
Not employed	58 555 (56.56)	(55.08)	(56.97)	.1071
Employed	38 423 (43.43)	(44.91)	(43.02)	
Exercised last month				
No	27 169 (31.06)	(29.05)	(31.61)	.0249
Yes	70 212 (68.93)	(70.94)	(68.38)	
Smoking history±				
Never smoked	52 771 (52.95)	(49.23)	(53.96)	<.001
Current smoker	12 952 (15.11)	(19.95)	(13.80)	
Former smoker	31 172 (31.92)	(30.81)	(32.23)	
Heavy drinking				
No	90 734 (94.49)	(94.14)	(94.59)	.3240
Yes	5011 (5.50)	(5.85)	(5.40)	
Experienced depression				
No	79 535 (82.67)	(77.79)	(84.00)	<.001
Yes	17 632 (17.32)	(22.20)	(15.99)	
BMI category				
Underweight (below 18.5)	1403 (1.44)	(1.54)	(1.41)	
Normal weight (18.5–24.9)	26 551 (27.98)	(26.98)	(28.26)	.6638
Overweight (25.0–29.9)	34 317 (37.53)	(38.05)	(37.38)	
Obese (30.0 and above)	29 054 (33.03)	(33.41)	(32.93)	
Chronic conditions				
No chronic condition	30 681 (34.49)	(30.91)	(35.46)	<.001
One chronic condition	30 222 (30.69)	(32.03)	(30.32)	
Two or more	34 708 (34.81)	(37.05)	(34.20)	
Fell at least once in the past year				
No	68 368 (74.34)	(70.01)	(75.52)	<.001
Yes	27 976 (25.65)	(29.98)	(24.47)	
Utilized dental services in the past year				
No	30 224 (34.43)	(35.21)	(34.21)	.3759
Yes	66 373 (65.56)	(64.78)	(65.78)	
Number of teeth removed				
No teeth removed	37 658 (38.55)	(47.65)	(53.55)	.1237
1–5 teeth removed	32 832 (34.85)	(32.90)	(29.80)	
6 or more teeth removed but not all teeth	15 423 (17.44)	(13.61)	(10.97)	
No teeth	8898 (9.14)	(5.81)	(5.66)	

^a^ Unweighted values.

^b^ Weighted percentages.

#### 3.1.2 Caregivers’ sociodemographic characteristics, health behaviors, and oral health status

Out of 20 089 caregivers aged 45 years or older, more than 65% of them were aged 45–64 years. Non‐Hispanic White caregivers accounted for 75%, 65.3% were married, and 33.4% had some college education (Table 1). About 55% of them were unemployed, nearly 71% had exercised in the past month, and 49% had never smoked. Furthermore, 78% of caregivers had experienced depressive disorders, about 38% were overweight, and 37% had two or more chronic conditions. When looking at the prevalence of falls among caregivers, around 30% of them fell at least once in the last year, which was higher than the prevalence of falls among non‐caregivers (24.5%, *p* ≤ .001). Most caregivers had used dental services in the past year, and there was no statistically significant difference between caregivers (64.8%) and non‐caregivers (65.8%). Furthermore, there was no statistically significant difference between caregivers and non‐caregivers in the number of teeth lost.

### 3.2 The association between oral health status, dental services utilization, caregiving status, and fall among U.S. respondents aged 45 years and older

The weighted logistic regression analysis presented in Table 2 assesses the impact of tooth loss and dental service utilization on the likelihood of falls among those aged 45 years and older, adjusted for caregiving status, socio‐demographic factors, physical activity, smoking history, alcohol drinking history, BMI, chronic diseases, and depression. Significant findings included a higher likelihood of falls with an increased number of teeth lost: those with one to five teeth removed had an AOR of 1.13 (95% CI: 1.02–1.24, *p* = .017), and the risk increased for those with six or more teeth removed (AOR: 1.28, 95% CI: 1.10–1.48, *p* = .001) and for complete tooth loss (AOR: 1.34, 95% CI: 1.14–1.57, *p* < .001). The analysis also indicated that individuals aged 45 years and older who had visited the dentist in the past year had 11.72% lower odds of falling compared to those who had not (AOR = 0.88, 95% CI: 0.79–0.97, *p* = .013). Additionally, caregivers had higher odds of falling compared to non‐caregivers (AOR: 1.23, 95% CI: 1.10–1.38, *p* < .001).

**TABLE 2:** Logistic regression for odds of falling at least once showing the association between oral health status, dental services utilization, caregiving status, and fall among U.S. respondents aged 45 years and older, BRFSS 2016, 2018, and 2020 (*N* = 97 550).^a^

Variables	AOR (95% CI)	*p*
**Number of teeth removed (Ref: No teeth removed)**		
1–5 teeth removed	1.13 (1.02–1.24)	**.017**
6 or more teeth removed but not all	1.28 (1.10–1.48)	**.001**
No teeth	1.34 (1.46–1.57)	**<.001**
**Dental services utilization (Ref: Did not visit dentist in the past 12 months)**		
Visited dentist in the past 12 months	0.88 (0.79–0.97)	**.013**
**Caregiving status (Ref: Non‐caregivers)**		
Caregiver	1.23 (1.10–1.38)	**<.001**

Bold values indicate statistically significant results (*p* < 0.05).

^a^ The model was adjusting for the number of teeth removed, dental services utilization, caregiver status, age, sex, marital status, education, employment status, physical activity, smoking, alcohol drinking history, BMI, number of chronic conditions, and depression.

### 3.3 The association between oral health status, dental services utilization, caregiving status, and fall among U.S. caregivers aged 45 years and older

The second weighted logistic regression analysis presented in Table 3 assesses the impact of tooth loss and dental service utilization on the likelihood of falls among caregivers aged 45 years and older, adjusted for socio‐demographic factors, physical activity, smoking, alcohol drinking history, BMI, chronic diseases, and depression. Our regression analysis shows that caregivers with six or more teeth removed had a 40% higher fall odds/prevalence (AOR: 1.40, 95% CI: 1.01–1.94, *p* = .038) and caregivers with all teeth removed had a 47% higher risk (AOR: 1.47, 95% CI:1.03–2.09, *p* = .030) compared to caregivers with no teeth removed. Dental service utilization was found to be a protective factor. Caregivers who visited the dentist in the past year had a significantly lower risk of falling (AOR = 0.79, 95% CI: 0.64–0.98, *p* = .035).

**TABLE 3:** Logistic regression for adjusted odds of falling at least once showing the association between oral health status, dental services utilization, and fall among U.S. caregivers aged 45 years and older, BRFSS 2016, 2018, and 2020 (*N* = 20 080).^a^

Variables	AOR (95% CI)	*p*
**Number of teeth removed (Ref: No teeth removed)**		
1–5 teeth removed	1.11 (0.90–1.36)	.313
6 or more teeth removed but not all	1.40 (1.01–1.94)	**.038**
No teeth	1.47 (1.03–2.09)	**.030**
**Dental services utilization (Ref: Did not visit dentist in the past 12 months)**		
Visited dentist in the past 12 months	0.79 (0.64–0.98)	**.035**

Bold values indicate statistically significant results (*p* < 0.05).

^a^ The model was adjusting for the number of permanent teeth removed, dental services utilization, age, sex, marital status, race/ethnicity, education, employment status, physical activity, smoking, alcohol drinking history, BMI, number of chronic conditions, and depression.

## 4 DISCUSSION

In this study, among a representative sample of middle‐aged and older adults in the US, oral health status and dental services utilization were associated with the prevalence of falls, even after accounting for caregiving status, demographic characteristics, behaviors, chronic conditions, and depression. This relationship was consistent among caregivers: those with tooth loss had higher odds of falling, and those who had received oral care in the past year had lower odds of falling. There is scant evidence in the literature on the relationship between the number of teeth removed, dental service utilization, and falls, especially among caregivers. A Malaysian study revealed a statistically significant association between the number of teeth present and the risk of falling.^7^ Similarly, a study conducted in Japan found that those with a low number of teeth present in their mouths had a significantly higher risk of falling than those who had lost less than 10 teeth.^6  ^Both study participants were aged 65 years and older, whereas our study included participants aged 45 years and older and caregivers and non‐caregivers. In addition, our study investigated dental service utilization and the prevalence of falls among caregivers and non‐caregivers, which to our knowledge had not been studied before. Our study found that visiting the dentist as recommended twice a year is a protective factor against experiencing a fall.

Consistent with the previous studies, our results showed that lost teeth were significantly associated with reporting falls.^6^, ^7^ In the regression, the likelihood of experiencing falls increased with more teeth lost, whereas those with no teeth were at the highest risk. Unlike previous studies, in this study, the sample population was further restricted to include only caregivers 45 years of age and older and demonstrated the same result. Given the known risks of negative health outcomes associated with caregiving, this finding highlights the importance of caregivers attending to their own health care, including regular oral health visits to prevent falls and the potential for fall‐related injuries.

The results highlighted that caregiving is associated with more reporting of falls. Caregivers had about 24% higher odds of falling at least once in the past year compared to non‐caregivers. This study appears to be one of a few to investigate such an association. Caregivers face significant physical burdens, including the need to lift and transfer care recipients and assist with daily living activities.^12^, ^25^, ^26^, ^27^ If one fails to follow proper body mechanics and safety precautions, these physically demanding tasks can increase the risk of falls. Additionally, the emotional stress of caregiving can lead to fatigue, anxiety, lack of attention, and depression, all of which impair cognitive function and physical performance, thereby increasing the likelihood of falls.^14^ Disrupted sleep patterns, often resulting from the care recipient's needs, further contribute to fatigue, decreased alertness,^13^, ^14^, ^15^ and an elevated risk of falls. Moreover, the time‐consuming nature of caregiving often leaves little opportunity for physical activity, leading to decreased strength and balance,^13^ which further heightens the risk of falls.

Poor oral health could exacerbate these risks. Caregivers may neglect their oral health, leading to conditions that contribute to systemic inflammation and physical discomfort, which may diminish balance and physical performance.^28^ These interconnected factors highlight the need for comprehensive care strategies that address both the physical and oral health of caregivers. Our results indicated that falls are more prevalent among individuals with poor oral health and those who do not visit the dentist regularly, particularly among caregivers. This is especially concerning as caregivers, who often neglect their own health, may face compounded risks. Previous studies have shown that caregivers, especially those with lower incomes, are less likely to seek preventive dental care, leading to complications such as tooth loss.^16^, ^17^ These findings support the growing body of literature that suggests poor oral health can increase the likelihood of falling. ^6^, ^7^, ^10^ This risk is increased for caregivers, who are already vulnerable due to the physical and emotional demands of caregiving, which can further degrade their quality of life.

To address the increased fall risk linked to caregivers' poor oral health and limited dental care access due to financial constraints, expanding Medicaid to include dental benefits is proposed. The American Dental Association has noted a decline in dental care utilization and low dentist participation in Medicaid, largely due to administrative burdens and inadequate reimbursement. Medicaid's varying coverage and high dental treatment costs further challenge access.^29^ Proposed interventions include establishing caregiver support programs for oral health, expanding dental insurance to cover essential treatments for those aged 45 years and older, and enhancing Medicaid to improve dental care access for low‐income individuals and caregivers, prioritizing dental health alongside general health.

Currently, fall prevention programs typically focus on balance and strength training, home safety assessments, and medication reviews for older adults.^30^ These programs should integrate dental health education into these frameworks to emphasize the potential connection between oral health and fall risks. Community and senior centers can offer these enhanced programs, which include home safety assessments for individuals with poor oral health. Furthermore, public awareness campaigns are essential for educating at‐risk groups, and policies should promote collaboration among health agencies and dental providers through task forces or strategic alliances to improve overall health outcomes.

Some limitations should be addressed. First, the BRFSS phone survey uses random‐digit dialing of landline and cell phones for non‐institutional households and therefore does not represent people living in institutional settings like nursing homes. Second, this research used self‐reported data. Recollection errors and other factors may have skewed the survey results. For example, tooth loss is hard to quantify, and people may forget earlier extractions. We do not expect that errors would have differed systematically by the respondents’ oral health or caregiving status, so this likely resulted in misclassification errors across all groups. Additionally, the BRFSS does not inquire about physical reasons or fall sites; it may not have collected enough information regarding participants' recent falls, therefore restricting the analysis on how oral health and caregiving status may contribute to fall prevalence. This study cannot establish cause‐and‐effect links due to cross‐sectional data; however, the use of large‐scale BRFSS data has the advantages of representing different demographic groups and a large and representative sample size. Finally, the study classified a diverse group of individuals as “on‐caregivers,” including those in need of care and former or future caregivers. This heterogeneity could dilute the distinction between caregivers and non‐caregivers, potentially affecting the association observed. Ideally, a more defined comparison group or longitudinal tracking of caregiving status could enhance clarity. However, due to BRFSS data limitations, this refinement was not feasible. Thus, while our comparison provides valuable initial insights, it requires cautious interpretation to avoid potentially underestimating the impact on caregivers.

Future longitudinal studies with adequate follow‐up time should test the effect of oral health status on fall and to investigate the relationship between the two and to examine how changes in one variable may affect the other variable over time. Future studies could research replacing missing teeth with removable prostheses, fixed prostheses, or dental implants and to determine which one could have better impact.

## 5 CONCLUSION

Poor oral health status in terms of teeth lost due to dental decay, gum disease, or infection is associated with higher odds of falling among individuals and caregivers aged 45 years and older. Conversely, more frequent dental care utilization is associated with lower odds of falling. This highlights oral health as a potential predictor of falls and suggests that providing frequent dental care and supporting oral health among middle‐aged and older adults could reduce falls, a leading cause of injury and disability in these age groups. Additionally, caregiver status is significantly associated with falls. Therefore, ensuring caregivers have access to preventive dental care could reduce their fall risk and, in turn, help ensure that their care recipients do not need additional or escalated care.

## AUTHOR CONTRIBUTIONS

All authors have contributed equally to all parts of the study. All authors read and approved the final manuscript.

## CONFLICT OF INTEREST STATEMENT

The authors have no conflict of interest to disclose.

The contents of this manuscript do not represent the views of the Department of Veterans Affairs or the United States Government.
